# Machine learning—enhanced baseline risk prediction models for cancer therapy related cardiac dysfunction: a systematic review and critical appraisal of methodological quality

**DOI:** 10.1093/ehjdh/ztag127

**Published:** 2026-08-10

**Authors:** Maria Sol Andres, Vijay Maharahan, Alexandra Pons Rivarola, Luis Garcia Rodriguez, Franck Thuny, Sivatharshini Ramalingam, Matilda Howe, Mamas A Mamas, Amitava Banerjee, Charlotte Manisty, Arjun K Ghosh, Aditya S Bharadwaj, Alexander R Lyon, Mohamed O Mohamed

**Affiliations:** Cardio-Oncology Service, Royal Brompton Hospital Centre of Excellence, Guy’s and St Thomas’ NHS Foundation Trust, Sydney Street, London SW3 6NP, UK; Cardio-Oncology Service, Royal Brompton Hospital Centre of Excellence, Guy’s and St Thomas’ NHS Foundation Trust, Sydney Street, London SW3 6NP, UK; Cardio-Oncology Service, Royal Brompton Hospital Centre of Excellence, Guy’s and St Thomas’ NHS Foundation Trust, Sydney Street, London SW3 6NP, UK; Cardio-Oncology Service, Royal Brompton Hospital Centre of Excellence, Guy’s and St Thomas’ NHS Foundation Trust, Sydney Street, London SW3 6NP, UK; Cardio-Oncology Service, Royal Brompton Hospital Centre of Excellence, Guy’s and St Thomas’ NHS Foundation Trust, Sydney Street, London SW3 6NP, UK; Aix-Marseille University, University Mediterranean Center of Cardio-Oncology, Unit of Heart Failure and Valvular Heart Diseases, Department of Cardiology, North Hospital, Assistance Publique, Marseille, France; Centre for CardioVascular and Nutrition Research (C2VN), Inserm 1263, Inrae 1260, Marseille, France; Cardio-Oncology Service, Royal Brompton Hospital Centre of Excellence, Guy’s and St Thomas’ NHS Foundation Trust, Sydney Street, London SW3 6NP, UK; Cardio-Oncology Service, Royal Brompton Hospital Centre of Excellence, Guy’s and St Thomas’ NHS Foundation Trust, Sydney Street, London SW3 6NP, UK; Keele Cardiovascular Research Group, Keele University, Keele, UK; Institute of Health Informatics, University College London, London, UK; Barts Heart Centre, Barts Health NHS Trust, London, UK; Barts Heart Centre, Barts Health NHS Trust, London, UK; Institute of Cardiovascular Science, University College London, London, UK; Barts Heart Centre, Barts Health NHS Trust, London, UK; Hatter Cardiovascular Institute, University College London Hospital, London, UK; Miller School of Medicine, University of Miami, Miami, USA; Cardio-Oncology Service, Royal Brompton Hospital Centre of Excellence, Guy’s and St Thomas’ NHS Foundation Trust, Sydney Street, London SW3 6NP, UK; Institute of Health Informatics, University College London, London, UK; Barts Heart Centre, Barts Health NHS Trust, London, UK

**Keywords:** Artificial intelligence, Machine learning, Cardiotoxicity, Cardiac dysfunction, Cardio-oncology, Risk prediction

## Abstract

**Aims:**

Cancer therapy-related cardiac dysfunction (CTRCD) is a prevalent complication with adverse clinical implications. Guidelines have emphasized the need for reliable baseline risk prediction to guide prevention strategies. While machine learning (ML) has shown promise in cardiovascular medicine, the readiness of ML-based prognostic models for baseline CTRCD risk prediction in cardio-oncology remains unclear.

**Aims:**

To systematically review and critically appraise the performance, methodological quality, and clinical applicability of ML-based models for predicting baseline CTRCD risk.

**Methods and results:**

A search on Embase and Medline was conducted up to May 2026. Eligible studies used ML algorithms to estimate CTRCD risk prior to cancer treatment. Data were extracted using an adapted version of the CHARMS checklist. Risk of bias (ROB) and reporting quality were assessed using PROBAST and TRIPOD + AI. Nine studies were included. Seven studies included novel models using multiple clinical and imaging predictors, while two repurposed or validated existing AI-enhanced electrocardiography (ECG) models. The levels of discrimination were at least moderate (AUC 0.65–0.88), calibration was only reported in two studies, and external validation in one. Multiparametric models showed higher performance metrics than ECG-only models but were methodologically constrained. Overall, most studies were limited by variability in outcome definitions, low event rates, limited transparency in reporting, and a lack of reproducibility measures.

**Conclusion:**

Current ML-based models for predicting CTRCD remain underdeveloped for clinical use. The present findings highlight the need for further work to optimize models by incorporating rigorous study designs, with emphasis on methodological clarity, standardized outcome definitions, and external validation to improve reproducibility.

## Graphical Abstract

**Graphical Abstract:**
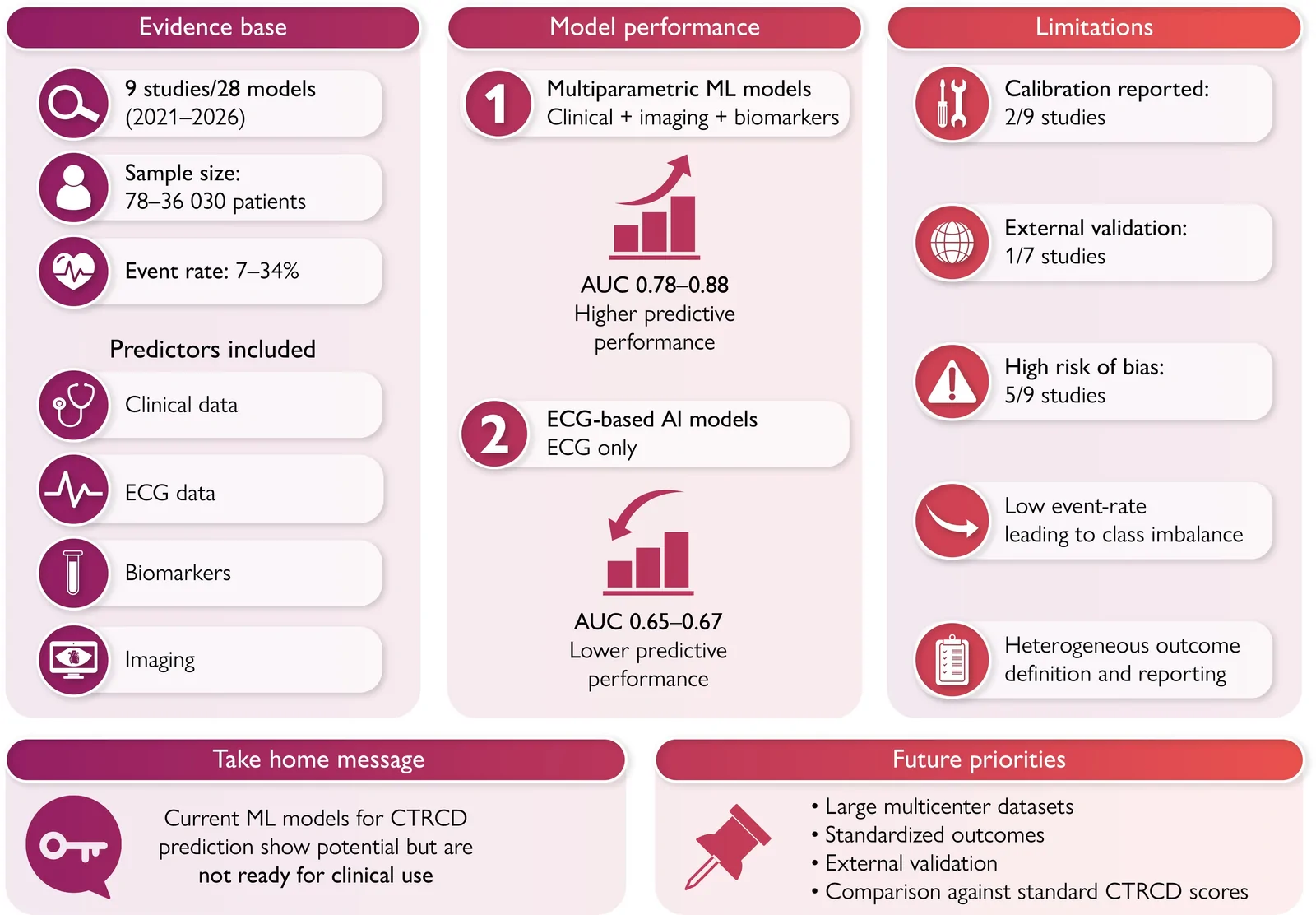

## Introduction

Cancer is one of the leading causes of morbidity and mortality worldwide.^1^ While systemic cancer therapies, including chemotherapy and targeted therapies, play a significant role in cancer management, they may adversely impact cardiac function, which is commonly defined as cancer therapy-related cardiac dysfunction (CTRCD).^2^ The incidence of clinically relevant CTRCD is estimated to be 9% among those undergoing anthracycline chemotherapy,^3^ approximately 16% with HER2-targeted therapies such as trastuzumab,^4^ and it has also been linked to various other cancer therapies.^5^ This can be highly consequential for cancer patients, resulting in discontinuation of life-saving cancer treatment and long-term cardiovascular (CV) health complications.

Despite advances in the diagnosis and management of CTRCD, there has been a lag in its primary prevention. The risk of cardiac toxicity is determined by many factors, including CV history, CV risk factors, oncological history and the type of cancer therapy.^6^ The use of predictive models to estimate the risk of developing CV events to assist and guide clinicians’ decision-making has become an area of growing interest. Major societies such as the European Society of Cardiology have identified this need in their cardio-oncology guidelines.^7^ However, existing tools have significant limitations in terms of validity. The Heart Failure Association (HFA) of the European Society of Cardiology and the International Cardio-Oncology Society (ICOS), published a joint position statement in 2020 outlining various predictive models for cardiotoxicity in patients scheduled to undergo cancer therapies. These models were not developed using statistical methodologies but were derived from expert opinion and literature review and had not been validated at the time of publication.^6^ The authors emphasized the importance of validation and recalibration to further optimize the proposed scores. A systematic review of risk prediction models based on traditional statistical modelling in patients with breast cancer was published in 2023.^8^ The evidence from six predictive models revealed methodological weaknesses throughout, leading to a high risk of bias and limiting the reliability of these tools in accurately predicting cardiotoxicity outcomes. This evidence was expanded in a recent review and meta-analysis encompassing 56 studies that either developed or validated models for CTRCD risk prediction. This study showed that most studies present a high risk of bias, few models underwent external validation and the HFA-ICOS risk score shows suboptimal performance.^9^ Finally, traditional cardiovascular risk scores, including the American Framingham score, the European Systematic Coronary Risk Evaluation (SCORE) 2 and the British QResearch Cardiovascular Risk Calculator (QRISK3), tend to systematically underestimate the cardiovascular risk in oncology patients as demonstrated by McCracken C. *et al.*^10^

While the use of machine learning (ML) has gained considerable momentum in cardiovascular risk prediction, the current evidence also suggests that many ML-based prediction models fail to generalize beyond their development datasets and often show reduced performance when externally validated.^11^ Important barriers to clinical implementation remain, including limited interpretability, incomplete reporting of calibration, substantial infrastructure requirements, and the paucity of prospective validation studies, which limit the real-world adoption of ML-based prediction tools in clinical practice.^11^

Nevertheless, several ML-based models have recently been developed to estimate the baseline risk of cancer therapy-related cardiac dysfunction. The present review sought to consolidate the evidence on these models and systematically assess their performance, methodological quality and clinical applicability in cardio-oncology.

## Methods

This systematic review was conducted in accordance with the Cochrane Collaboration guidelines following the Preferred Reporting Items for Systematic Reviews and Meta-Analyses (PRISMA) statement.^12^ The study protocol review was registered in the International Prospective Register of Systematic Reviews, PROSPERO (CRD420251013899) in March 2025 and last edited on the 27 February 2026.^13^

### Literature search strategy

A literature search was conducted through the Ovid platform in the Embase and Medline libraries, with no publication date restriction, on the 22 May 2026. Additional manual searches were conducted using reference lists of included studies. Key concepts from the research question were identified and combined into a comprehensive search strategy. The combined search terms are summarized below:

(‘artificial intelligence’ OR ‘machine learning’ OR ‘AI augmentation’ OR ‘deep learning’ OR ‘AI’) AND (‘cardiooncology’ OR ‘cardio-oncology’ OR ‘cardiotoxicity’ OR ‘cardiovascular toxicity’ OR ‘cardiac toxicity’ OR ‘toxicity’ OR ‘dysfunction’) AND (‘cardiac’ OR ‘cardiovascular’ OR ‘heart’) AND (‘cancer’ OR ‘malignancy’ OR ‘oncology’) AND (‘chemotherapy’ OR ‘anthracycline’ OR ‘cytotoxic regimens’ OR ‘immunotherapy’ OR ‘cancer treatment’ OR ‘therapy-induced’) AND (‘risk’ OR ‘prediction’ OR ‘risk prediction’ OR ‘model’ OR ‘classifier’ OR ‘cardiac risk’ OR ‘risk stratification’).

The full search strategy is outlined in the Supplementary material (Supplementary material online, *Table S1*).

### Selection of studies

The result of the search was split among six independent reviewers (MSA, VM, MH, AP, LR, FT), such that each paper was assessed by at least two reviewers for eligibility to our study. Any discrepancies were resolved through discussion and consensus between the two reviewers; if consensus could not be reached, a third reviewer, blinded to the prior decisions, provided the final judgment.

### Eligibility criteria and study selection

The PICOT (population, intervention, comparator, outcome, timing) framework was used to define the eligibility criteria.

- Population: Patients with a diagnosis of cancer who need systemic cancer treatment.
- Intervention (model): Models that use at least one ML algorithm to predict the baseline risk of CTRCD.
- Control: not applicable.
- Outcomes of interest: CTRCD as defined by the International Cardio-Oncology Society^2^ including symptomatic or asymptomatic left ventricular dysfunction, heart failure, and cardiomyopathy.
- Time: event occurrence at any time after chemotherapy commencement.

Overall, this systematic review included research studies involving patients diagnosed with cancer, undergoing systemic cancer therapy, where the incidence of cardiovascular outcomes, including any degree of CTRCD, was reported either as a primary or a secondary outcome. Eligible studies must have utilized at least one ML algorithm to estimate the risk of this event before systemic cancer therapy was commenced (baseline risk). A comprehensive list of the inclusion and exclusion criteria is presented in *Table 1*. Studies identified through the literature search strategy were screened for eligibility criteria at the abstract level using bibliographic software (Covidence) to facilitate collaboration among reviewers. Full texts of the provisionally eligible studies were further examined, which also enabled identification of additional relevant studies from the references.

**Table 1:** Eligibility criteria for the systematic review

Criteria	Details
**Inclusion criteria**	Original research papers Papers that used at least one ML algorithm to predict risk of CTRCD. The incidence of CTRCD, as defined by the ICOS, was reported either as a primary or a secondary outcome. ML models and predictor variables were clearly described. Reported the performance of the model in terms of AUC, PPV, NPV, Sensitivity, Specificity, Accuracy, Precision or F1-score (at least one of these parameters).
**Exclusion criteria**	Conference abstracts, letters, editorials, systematic reviews or meta-analyses, consensus statements, and guidelines. Risk scores built exclusively on traditional statistical methods. Heart failure, cardiac dysfunction, or CTRCD not included in the endpoints. Studies for which the full text is not available.

AI, artificial intelligence; CTRCD, cancer therapy-related cardiac dysfunction; ICOS, International Cardio-Oncology Society; ML, machine learning; AUC, area under the curve; PPV, positive predictive value; NPV, negative predictive value.

### Data extraction and synthesis

Prespecified information was extracted independently and in duplicate from the included studies using a standardized data extraction form to ensure consistency and minimize errors. An adaptation of the Checklist for Critical Appraisal and Data Extraction for Systematic Reviews of Prediction Modelling Studies (CHARMS) was used for this purpose and is presented in Supplementary material online, *Table S2*. Meta-analysis of comparative data was not possible due to the limited number of studies available, heterogeneity in models and outcome definitions, and a lack of published data on the variance of outcome measures.

### Risk of bias assessment and reporting transparency assessment

Assessment of the risk of bias (ROB) of the individual studies was performed using the Prediction model Risk of Bias Assessment Tool (PROBAST). Data was extracted to complete the four PROBAST domains: study participants, predictors, outcome, and analysis. For each domain, the tool provides signalling questions to determine whether the ROB should be graded as low, high, or unclear.^14^ The reviewers recorded the ROB for each study item, with discrepancies resolved by consensus. The overall ROB was summarized in a ‘traffic light plot’. We used the Transparent Reporting of a Multivariable Prediction Model for Individual Prognosis or Diagnosis-Artificial Intelligence (TRIPOD + AI) reporting guideline to assess consistency in reporting of the included studies.^15^

## Results

**Figure 1:**
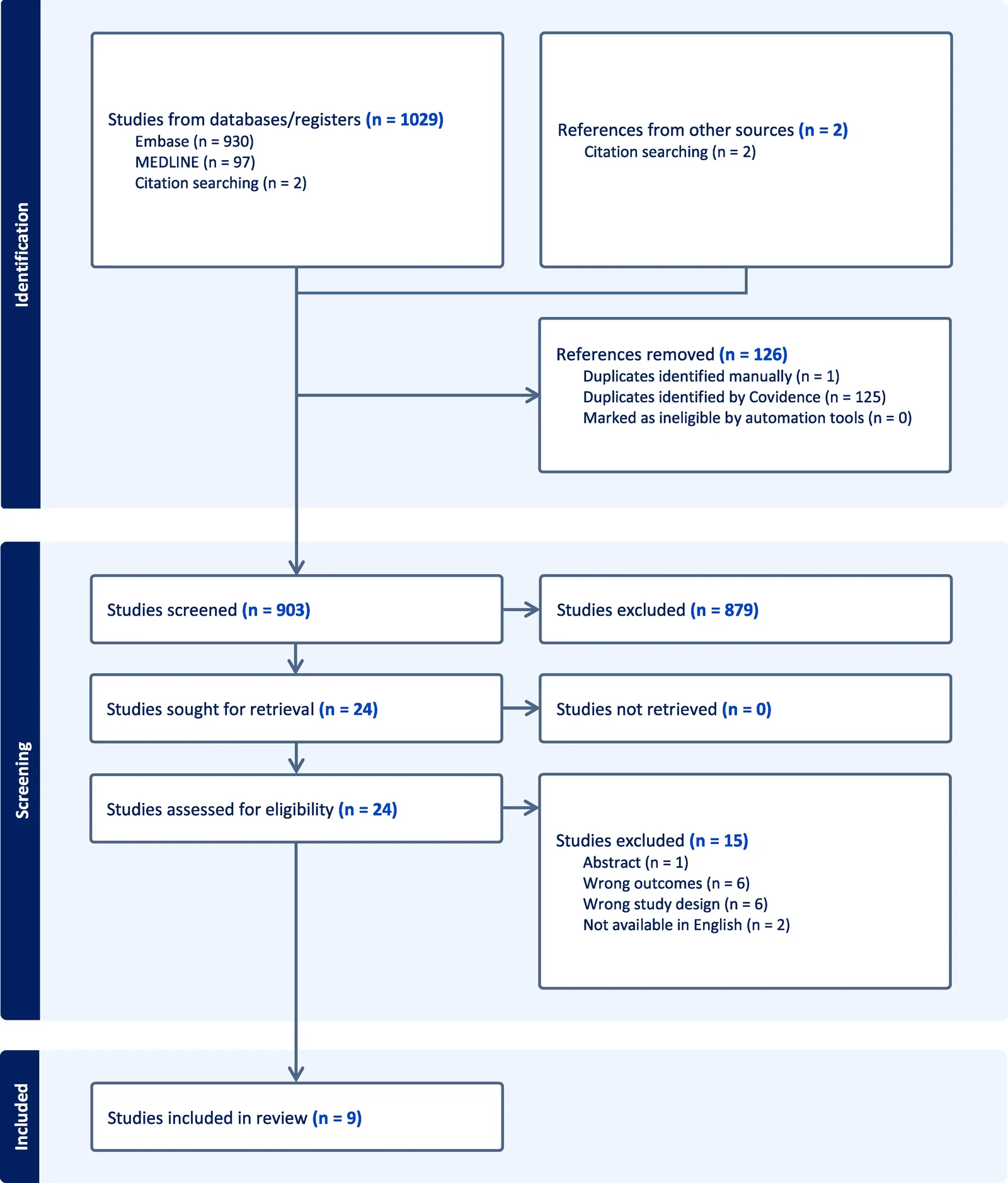
PRISMA flowchart of study inclusion

The search identified 1029 studies; 930 were retrieved from Embase, 97 from Medline, and 2 were manually added through citation searching. One hundred and twenty-six studies identified as duplicates by Covidence and one identified manually were removed, leaving 903 studies for title and abstract screening. After the removal of 879 studies deemed irrelevant, 24 studies were identified for full-text assessment. Of these, we included nine references for the final analysis.^16–24^ The PRISMA flowchart of the search strategy and selection process is illustrated in *Figure 1*.

### Development and validations studies

**Figure 2:**
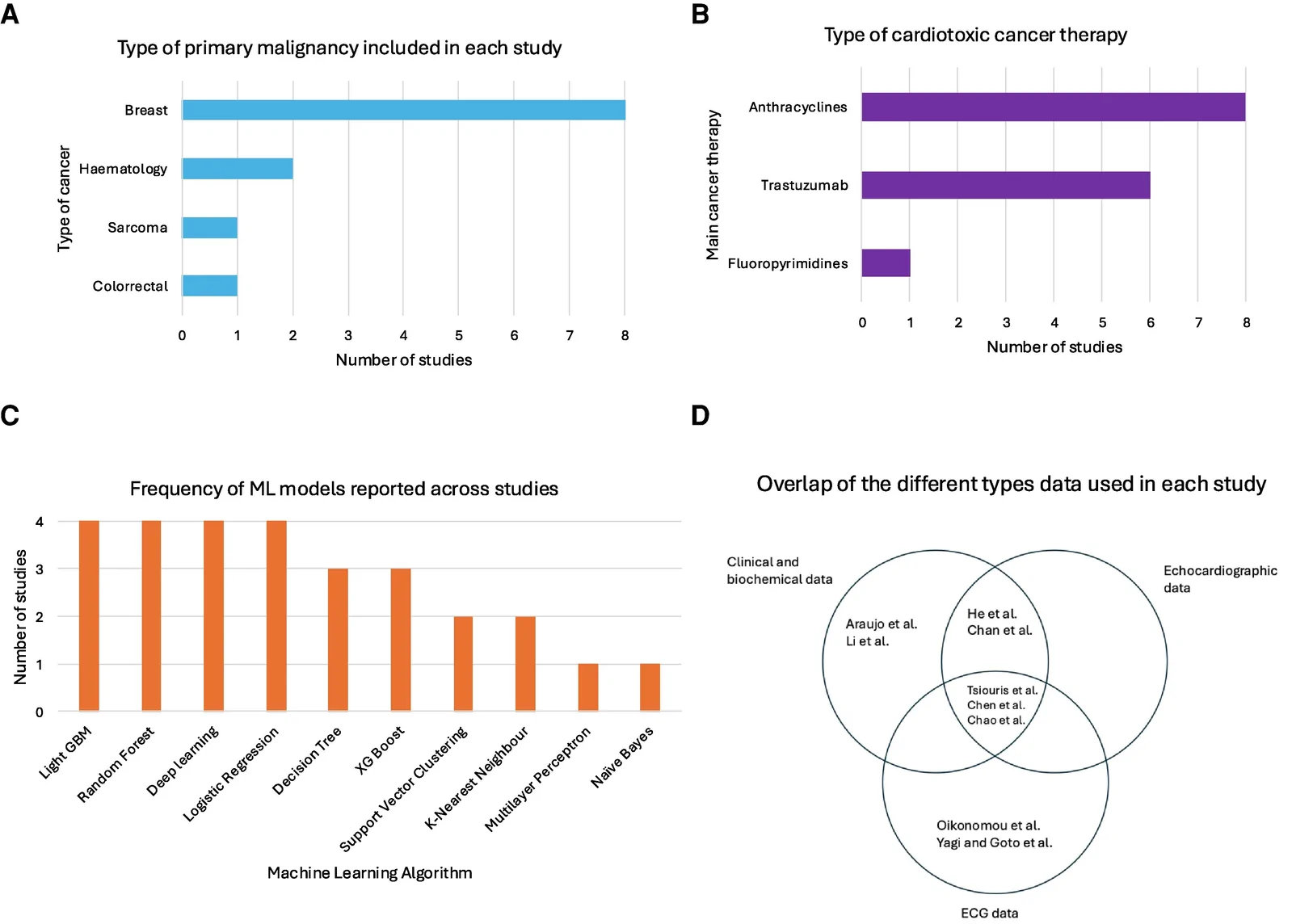
Characteristics of the included studies. **(***A*) Type of primary malignancy diagnosed in the patients included in each study. (*B*) Type of cardiotoxic cancer therapy included. (*C*) Frequency of Machine Learning models reported across the 9 studies and 28 models. (*D*) Different types of data used as predictor variables in each study,

Among the nine studies included, seven focused on developing new machine learning models,^18–24^ one applied transfer learning from an existing model,^17^ and one validated a previously developed model in a different population and for a distinct yet relevant outcome.^16^ Of the seven studies that included newly developed models, only two reported calibration metrics^23,24^ and one external validation data.^19^ Notably, the two studies that leveraged existing models explored the use of artificial intelligence-enhanced electrocardiography (ECG) to predict baseline CTRCD risk. In contrast, the remaining seven studies designed models based on a combination of clinical, demographic, laboratory, and imaging data (*Figure 2D*). In this systematic review, models based on ECG inputs will be referred to as *ECG-based models*. Those incorporating broader predictor variables will be referred to as *clinical predictor-based models*.

### Clinical predictor-based models

Chen *et al*., 2026,^23^ Cao *et al*., 2026,^24^ He *et al*., 2025,^21^ Araujo *et al*., 2025,^22^ Tsiouris *et al*., 2023,^18^ Chang *et al*., 2022 ,^19^ Li *et al*., 2022 ^20^

### Participants

These seven studies evaluated newly developed models for baseline risk prediction of CTRCD. Five used retrospective cohort data.^18,20,21,23,24^ The sample size ranged from 270 to 36 030 participants. The remaining two studies^19,22^ were prospective, single-centre cohorts with smaller sample sizes of 78 and 211 patients(*Table 2*). Six studies focused on breast cancer populations and aimed to predict the risk of CTRCD associated with anthracyclines and/or anti-Human Epidermal Receptor 2 (HER2) therapies.^18,19,21,22^ The seventh study included patients with colorectal cancer under fluoropyrimidine treatment (*Figure 2A* and *B*). Reported mean ages ranged from 50 to 72 years; one study did not provide age data.

**Table 2:** Characteristics of machine learning-based baseline risk models

Study characteristics		Participants characteristics				
Author, year	Source of data	No. of patients	Age, y	Sex (female)	Type of cancer	Systemic treatment
**Chen *et al.*, 2026^23^**	Retrospective cohort study.	423	Mean: 50.9, SD 9.54	100%	Breast Cancer	Anthracyclines, Trastuzumab
**Cao *et al.*, 2026^24^**	Retrospective cohort study, China, single centre.	2034	Mean: 54.8, SD:10.5	100%	Breast Cancer	Anthracyclines (100%) Trastuzumab (12.5%)
**He *et al.*, 2025^21^**	Retrospective cohort study, Piñero Lamas *et al.* dataset, single centre.	270	Median: 65, IQR: 45–65^a^	100%	Breast cancer (HER2+)	Anthracyclines, Trastuzumab
**Araujo *et al.*, 2025^22^**	Prospective cohort study, Brazil, single centre.	78 patients (4892 synthetic samples)	Mean: 50, SD: 12.8	100%	Breast cancer	Anthracyclines (100%)
**Tsiouris *et al.*, 2023^18^**	Retrospective cohort study, CARDIOCARE project dataset, multicentre.	1560	NR^b^	NR^b^	Breast cancer	Anthracyclines (68.7%), Trastuzumab (71.2%)
**Chang *et al.*, 2022^19^**	Prospective cohort study, Taiwan, single centre.	211	Mean: 58, SD: 10.28	NR	Breast cancer	Anthracyclines (100%), Trastuzumab (25.6%)
**Li *et al.*, 2022^20^**	Retrospective cohort study, SEER-Medicare database, multicentre.	36030	Mean: 72, SD: 9.19	47%	Colorectal cancer	Fluoropyrimidines-based chemotherapy (100%)
**Oikonomou *et al.*, 2025** ^16^	Retrospective cohort study, Yale New Haven Health System, multicentre.	1550	Median: 60, IQR: 51–69	79%	Breast cancer (63.4%), Non-Hodgkin Lymphoma (36.6%)	Anthracyclines (79.7%), Trastuzumab (24.5%)
**Yagi and Goto *et al.*, 2024** ^17^	Retrospective cohort study, BWH/MGH and Keio University Hospital registry data.	1328	Mean: 57.5, SD: 16.7	52%	Lymphoma (35.2%), Leukaemia (33.8%), Breast cancer (13.6%), Sarcoma (9.6%), other (7.9%)	Anthracyclines (100%)

NR, not reported; SD, standard deviation; IQR, interquartile range; HER2+, human epidermal growth factor receptor positive; 5-FU, 5-fluorouracil; BWH, Brigham and Women Hospital; MGH, Massachusetts General Hospital.

^a^The median age reported corresponds to the original cohort of 531 patients reported in the Piñero Lamas *et al.* dataset (Piñero-Lamas B *et al*. A cardiotoxicity dataset for breast cancer patients. Sci Data. 2023;10(1):527). The median age for the cohort of 270 patients utilized for this study was not reported on the published manuscript.

^b^The raw data of the CARDIOCARE database is not available to the public.

### Machine learning models and predictors

Three studies assessed the performance of a single ML algorithm within their models; He *et al*.^21^ used deep learning, Araujo *et al.*^22^ used Light Gradient Boosting Machine while Tsiouris *et al*.^18^ used a decision tree. The remaining four studies implemented multiple ML algorithms and compared their predictive performances^19,20,23,24^ (*Table 3*). The number of predictors used across the five models ranged from 5 to 111. All studies incorporated clinical predictors, including demographic characteristics, cardiovascular risk factors, cancer-related variables, medications, and other comorbidities. Two studies integrated clinical data with echocardiographic reports,^19,21^ one combined clinical and laboratory data,^22^ and three incorporated three types of data: clinical, imaging, and biochemical^18,23,24^ (*Figure 2D*). A detailed summary of the predictors used in each study is provided in the Supplementary material (Supplementary material online, *Table S3*).

**Table 3:** Machine learning models development and validation stage

Author, year	ML-model	Predictors used in the final model	Internal validation	Calibration	External validation
**Chen *et al.*, 2026 ^23^**	Logistic Regression, Decision Tree, Random Forest, XGBoost, Support Vector Machine, K-nearest neighbour, NB and Light GBM	Baseline Echocardiographic data (LVEF) + abnormal ECG findings + clinical/demographic data (Age, chemotherapy)	Yes (training data set: 70%, testing data set: 30%)	Yes (Calibration plots and Brier score)	No
**Cao *et al.*, 2026^24^**	Logistic Regression, Decision Tree, Random Forest, GBM, XGBoost, TabNet (Deep Learning)	Baseline clinical/demographic data + echocardiography data + laboratory data + ECG data	Yes (training data set: 80%, testing data set: 20%)	Yes (Calibration plots and Hosmer-Lemeshow goodness-of-fit test)	No
**He *et al.*, 2025^21^**	Deep Learning—TPNET	Baseline Echocardiography data (TDI and LV function) + clinical data	Yes (five-fold cross validation)	No	No
**Araujo *et al.*, 2025^22^**	Light GBM	Baseline laboratory biomarkers + clinical/demographic data + engineered features	Yes (training data set: synthetic data from 78 patients with data augmentation smoothing, test data set: real data)	No	No
**Tsiouris *et al.*, 2023^18^**	Decision tree	Baseline clinical/demographic data + echocardiography data + laboratory data	Yes (training data set: 60%, testing data set: 40%)	No	No
**Chang *et al.*, 2022^19^**	Random Forest, Logistic Regression, Support Vector Clustering, Light GBM, K-nearest neighbour, Multilayer perceptron	Baseline clinical/demographic data + echocardiography data (LVEF)	Yes (training data set: 70%, testing data set: 30%)	No	Yes (*n* = 16)
**Li *et al.*, 2022^20^**	XG Boost, Random Forest, Logistic Regression	Baseline clinical/demographic data	Yes (training data set: 2006–2011 data, testing data set: 2012–2014, ratio 2:1 for training and testing)	No	No
**Oikonomou *et al.*, 2025^16^**	Deep Learning - EfficientNet B3 CNN)	Baseline 12 lead ECG	No (validation study)	No	Yes
**Yagi and Goto *et al.*, 2024^17^**	Deep Learning - 2D CNN	Baseline 12 lead ECG	Yes (training data set: 23.9%, testing data set: 76.1%)	No	No

IQR, interquartile range; TPNET, temporal multimodal pattern network for efficient training; GBM, gradient boosting machine; XGBoost , extreme gradient boosting; CNN, convolutional neural network.

**Figure 3:**
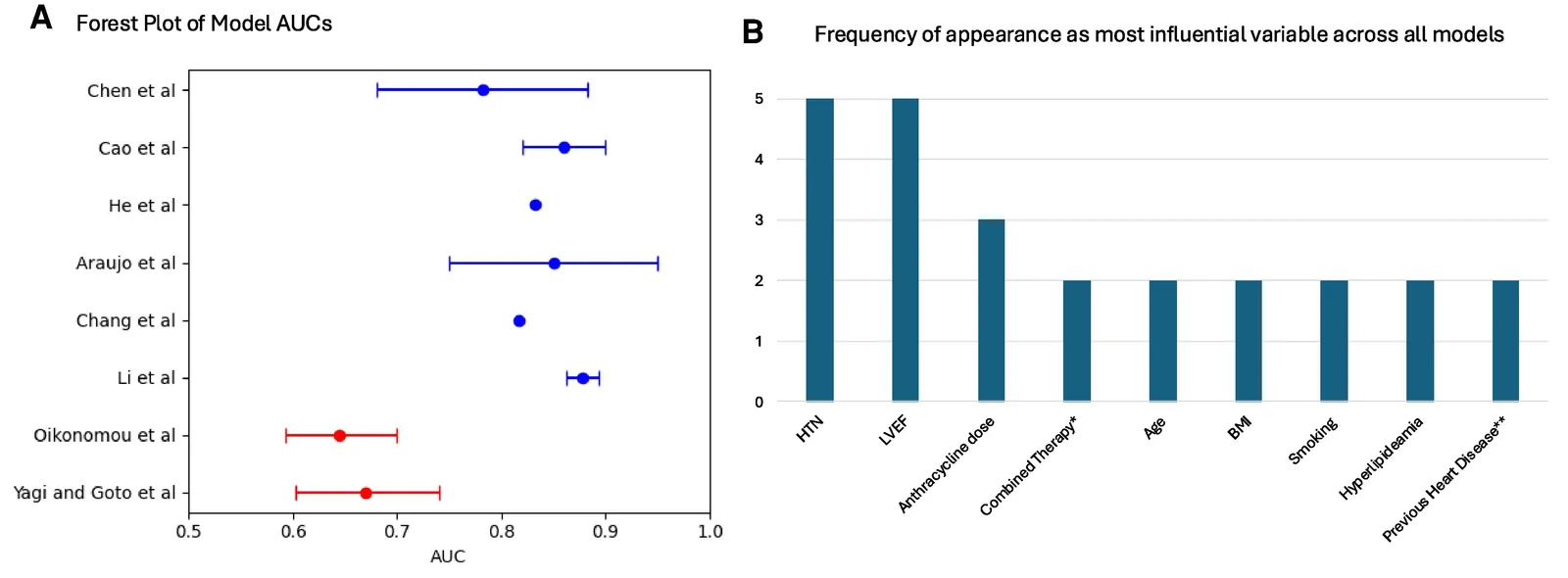
Summary of the reported results. (*A*) Forest plot of the area under the receivers operator characteristics curve reported for each study and the confidence intervals (CI) wherever reported. Tsiouris *et al.*’s^18^ model is not included in this graph because an AUC was not reported. He *et al.*^21^ and Chang *et al.*^19^ are represented as a dot without distribution bars because the CI were not reported. If the study included more than one ML model, the best-performing algorithm is represented in this forest plot. **(***B***)** Bargraph showing the frequency of appearance as the most influential variable across all models. Each study identified and reported the most impactful predictors influencing their model performance. Of the clinical features, hypertension and LVEF emerged as the most frequently significant variable.

Each study identified and reported the most impactful predictors influencing their model performance. Hypertension emerged as the most frequently significant variable present in five out of seven studies, followed by anthracycline dose, combined therapy (anthracyclines and trastuzumab), age, body mass index, and smoking. Pre-existing heart disease, including coronary artery disease and cardiomyopathy, was identified as a strong predictor in two out of four studies that did not exclude patients with such a condition. Dyslipidaemia was found to be an influential variable in two different studies, although Araujo *et al*.^22^ ‘s model identified an inverse effect between total cholesterol (lower values predicted a higher risk of cardiotoxicity) and the LDL/HDL ratio (higher values predicted a higher likelihood of CTRCD). Additionally, in all five studies that included baseline echocardiographic data, left ventricular ejection fraction (LVEF) was a key determinant (*Figure 3B*).

### Outcome definition and follow-up duration

The outcome of interest was defined as a reduction in LVEF of more than 10% from baseline in three of the breast cancer studies (*Table 4*).^18,19,21^ The definition of CTRCD included an LVEF drop of 5% if heart failure symptoms were present or a change in longitudinal strain from baseline in one study^24^ and new or worsened ECG abnormalities in another case.^23^ Finally, one study provided a more ambiguous definition, referring only to ‘myocardial dysfunction’ without further clarification.^22^ Follow-up durations in these breast cancer studies ranged from 1–3 years and two studies did not provide information regarding follow-up periods or timeframe for outcome identification. In contrast, the colorectal cancer study defined cardiotoxicity as a cardiovascular event within 30 days of treatment, including ischaemic heart disease, heart failure, cardiomyopathy, arrhythmia, acute myocarditis, stroke, cardiogenic shock, and sudden cardiac arrest.^20^ The authors also conducted sensitivity analyses using three ML algorithms with a more restricted definition limited to heart failure and cardiomyopathy. This narrower definition better aligned with the review’s focus, which is why the study was included in the final analysis.

**Table 4:** Machine learning models outcomes and performance metrics

Author, year	Outcome definition	Outcome event rate, *n* (%)	Follow-up time	AUC-ROC	Sensitivity	Specificity	Accuracy	F1 score
**Chen *et al.*, 2026** ^23^	**CTRCD:** Drop in LVEF >10% to below 53% from baseline, or new ECG abnormalities, or biomarker elevation	All events: 111 (26.4%)	Not reported	LR: 0.77	LR: 0.714	LR: 0.674	LR: 0.694	LR: 0.857
				DT: 0.627	DT: 0.593	DT: 0.804	DT: 0.698	DT: 0.516
		LVEF drop or HF symptoms: 57 (13.4%)		RF: 0.751	RF: 0.714	RF: 0.798	RF: 0.756	RF: 0.641
				XGB: 0.782	XGB: 0.657	XGB: 0.831	XGB: 0.744	XGB: 0.698
				SVM: 0.778	SVM: 0.8	SVM: 0.596	SVM: 0.698	SVM: 0.566
				KNN: 0.728	KNN: 0.429	KNN: 0.82	KNN: 0.624	KNN: 0.455
				NB: 0.674	NB: 0.486	NB: 0.854	NB: 0.67	NB: 0.523
				LGBM: 0.506	LGBM: 0.657	LGBM: 0.326	LGBM: 0.491	LGBM: 0.39
**Cao *et al.*, 2026^24^**	**CTRCD:** LVEF drop ≥ 10% to 53% or ≥5% + HF symptoms, or GLS drop > 15%, or new biomarker elevation	305 (15%)	Not reported	LR: 0.66	LR: 0.68	LR: 0.64	NR	NR
				DT: 0.66	DT: 0.67	DT: 0.67		
				RF: 0.75	RF: 0.78	RF: 0.78		
				GBM: 0.75	GBM: 0.80	GBM: 0.80		
				XGB: 0.79	XGB: 0.82	XGB: 0.82		
				TN: 0.86	TN: 0.85	TN: 0.85		
**He *et al.*, 2025^21^**	LVEF drop below 50% and by at least 10 points from baseline	27 (10%)	Median (IQR): 518 days (278–1146)	0.832	0.88	0.73	NR	NR
**Araujo *et al.*, 2025^22^**	New myocardial dysfunction	27 (34.6%)	12 months	0.85	0.89	0.69	0.76	0.72
**Tsiouris *et al.*, 2023^18^**	LVEF decline ≥10% from baseline (by echocardiography)	48 (15.1%)	12 months	NR	0.73	NR	0.7	0.71
**Chang *et al.*, 2022 ^19^ (CTRCD, development)**	**CTRCD:** Drop in LVEF >10% to below 50% from baseline	23 (10.9%),	3 years	RF: 0.491	RF: 0	RF: 0.982	RF: 0.875	RF: 0.467
				LR: 0.558	LR: 0.571	LR: 0.544	LR: 0.547	LR: 0.449.
				SVM: 0.584	SVM: 0.571	SVM: 0.596	SVM: 0.594	SVM: 0.479
				KNN: 0.495	KNN: 0.429	KNN: 0.561	KNN: 0.547	KNN: 0.430
				LGMB: 0.563	LGMB: 0.143	LGMB: 0.982	LGMB: 0.891	LGMB: 0.582
				MLP: 0.664	MLP: 0.857	MLP: 0.526	MLP: 0.562	MLP: 0.491
**Chang *et al.*, 2022 (HFrEF, development)**	**HFrEF:** LVEF <40% and symptoms of heart failure	8 (3.8%)	3 years	RF: 0.500	RF: 0	RF: 1	RF: 0.969	RF: 0.492
				LR: 0.823	LR: 1	LR: 0.645	LR: 0.656	LR: 0.469
				SVM: 0.806	SVM: 1	SVM: 0.613	SVM: 0.625	SVM: 0.451
				KNN: 0.411	KNN: 0	KNN: 0.823	KNN: 0.797	KNN: 0.443
				LGMB: 0.500	LGMB: 0	LGMB: 1	LGMB: 0.969	LGMB: 0.492
				MLP: 0.790	MLP: 1	MLP: 0.726	MLP: 0.734	MLP: 0.516
**Chang *et al.*, 2022 (HFrEF, validation)**		NR	NR	0.817	0.833	0.8	0.813	0.806
**Li *et al.*, 2022^20^**	CTR-CVT: any cardiotoxicity event within 30 days of starting treatment.	All CV events: 6753 (18.7%) HF: NR	30 days	HF XG: 0.878 LR: 0.877 RF: 0.862	NR	NR	NR	HF XG: 0.177 LR: 0.191 RF: 0.044
	Sensitivity analysis: HF or cardiomyopathy							
**Oikonomou *et al.*, 2025^16^**	1)Diagnostic code for cardiomyopathy or heart failure and	238 (15.4%)	12 months	0.645	NR	NR	NR	NR
	2) LVEF drop to below 50%							
**Yagi and Goto *et al.*, 2024^17^**	LVEF drop below 53% and by at least 10 points from baseline	106 (7.9%)	Median (IQR) 560 days (149–999)	AI-CTRCD model: 0.67 w/Clinical Data: 0.78	0.72	0.43	0.46	0.21

NR, not reported. TDI, tissular Doppler imaging; LV, left ventricle; LVEF, left ventricular ejection fraction; ECG, electrocardiogram; CTRCD, cancer therapy cardiac dysfunction; CTR-CVT, cancer therapy related-cardiovascular toxicity; HF, heart failure; AUC-ROC, area under the receiver operating characteristic; LR, logistic regression; DT, decision tree; RF, random forest; XGB, extreme gradient boosting; SVM, support vector machine; KNN, K-nearest neighbour; NB, Naïve Bayes; TN, Tab Net (deep learning); LGBM, light gradient boosting machine); MLP, multilayer perceptron.

### Performance

The reported performance metrics are summarized in *Table 4* and shown in *Figure 3A*. Six studies reported model discrimination performance using the area under the receiver operating characteristic curve (AUC), although two did not report the corresponding confidence intervals. In four of these, the AUC exceeded 0.80, indicating high discriminatory ability.^20–22,24^ Chang *et al*.^19^ evaluated six ML algorithms and found that the multilayer perceptron (MLP) yielded the best performance, with AUCs of 0.66 for predicting CTRCD, 0.79 for heart failure with reduced ejection fraction below 40%, and 0.817 in an external validation cohort including 16 patients.^19^ Tsiouris *et al*.^18^ did not report the AUC but instead highlighted the model's high sensitivity (73.45%), along with an accuracy of 70%, precision of 71%, and an F1-score of 71%.^18^ Only two of the included studies reported calibration metrics.^23,24^

### ECG-based models

Oikonomou *et al*., 2025,^16^ Yagi and Goto *et al*., 2024,^17^ Two studies employed baseline ECGs as the sole input for CTRCD prediction, leveraging previously developed deep learning algorithms. Their characteristics are presented in *Table 2*. Oikonomou *et al*.^16^ utilized a retrospective cohort to apply a validated AI-ECG model, originally trained using echocardiographic labels to detect left ventricular systolic dysfunction.^16^ The objective was to assess whether this model could identify patients at elevated risk of CTRCD. The study included 1550 patients (median age: 60 years) who had been treated with anthracyclines or trastuzumab. The model was tested on the entire cohort without additional training. It employed an EfficientNet B3 convolutional neural network, and the tool is available to end users via personal computers or handheld devices.

Similarly, Yagi and Goto *et al*.^17^ repurposed a previously trained deep learning model, initially designed to detect reduced LVEF from ECGs, using a transfer learning approach to predict CTRCD prior to chemotherapy. Their retrospective dataset included 1328 patients. Of these, 317 patients were allocated to the training set, and the remaining 1011 to the test set.^17^

Outcome definitions and adjudication differed between studies. One study defined CTRCD using a combination of clinical codes and echocardiographic reports,^16^ whereas the other relied exclusively on echocardiography,^17^ despite follow-up imaging being unavailable for 42.5% of the cohort. The LVEF threshold was also different in both studies. The predictive performance of both models was moderate (*Table 4*, *Figure 3A*), with AUC values of 0.645 and 0.672, respectively. Yagi and Goto *et al*.^17^ also evaluated a hybrid model that combined their AI-based CTRCD prediction with conventional cardiovascular risk factors. This combined approach improved the AUC to 0.78.

### Risk of bias and transparent reporting assessment among the included studies

**Figure 4:**
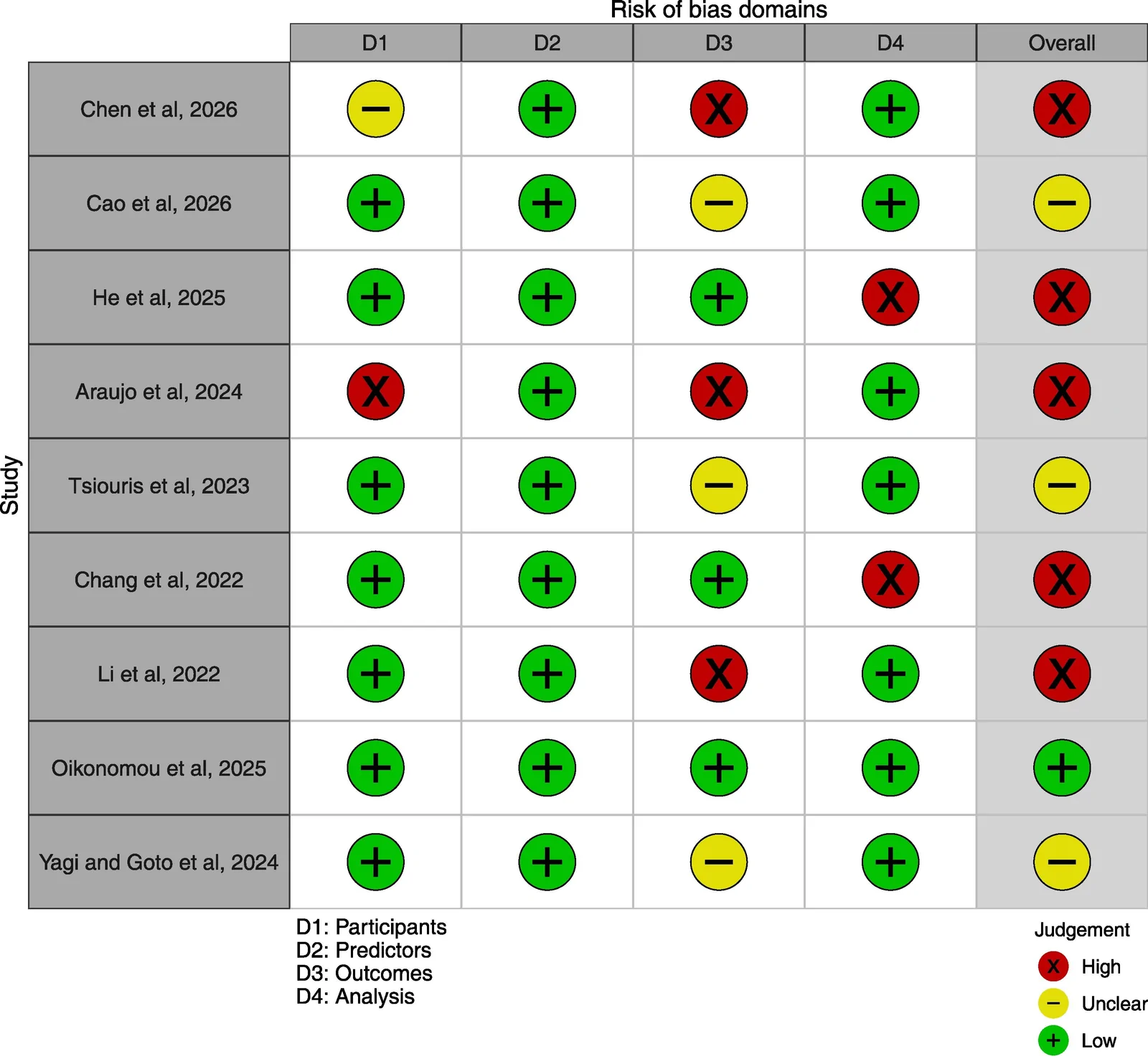
PROBAST risk of bias assessment results of the nine studies. Domains: D1: Participants—Assesses bias due to participant selection, recruitment, or inclusion/exclusion criteria. D2: Predictors—Evaluates bias arising from the measurement or classification of predictors. D3: Outcomes—Assesses bias in outcome measurement, including objectivity and measurement consistency. D4: Analysis—Evaluates bias in statistical analyses, including handling of missing data, model appropriateness, and selective reporting. Overall—Summary risk of bias considering all domains. Judgment categories: Low (green ‘+’): Low risk of bias; methods and reporting are appropriate and unlikely to meaningfully distort results. Unclear (yellow ‘–’) : Risk of bias is uncertain due to incomplete information or methodological ambiguity. High (red ‘×’): High risk of bias; flaws in study design, conduct, or reporting are likely to distort results.

According to the PROBAST assessment, five studies were rated as having a high risk of bias,^19–23^ three studies were rated unclear,^17,18,24^ and one as low risk of bias^16^(*Figure 4*). Sources of bias included imprecise outcome definitions (6/9), insufficiently described handling of missing data (1/9), and substantial manipulation of small or imbalanced datasets through data-augmentation techniques that were not subsequently evaluated in external cohorts (2/9), raising concerns about potential overfitting.

**Figure 5:**
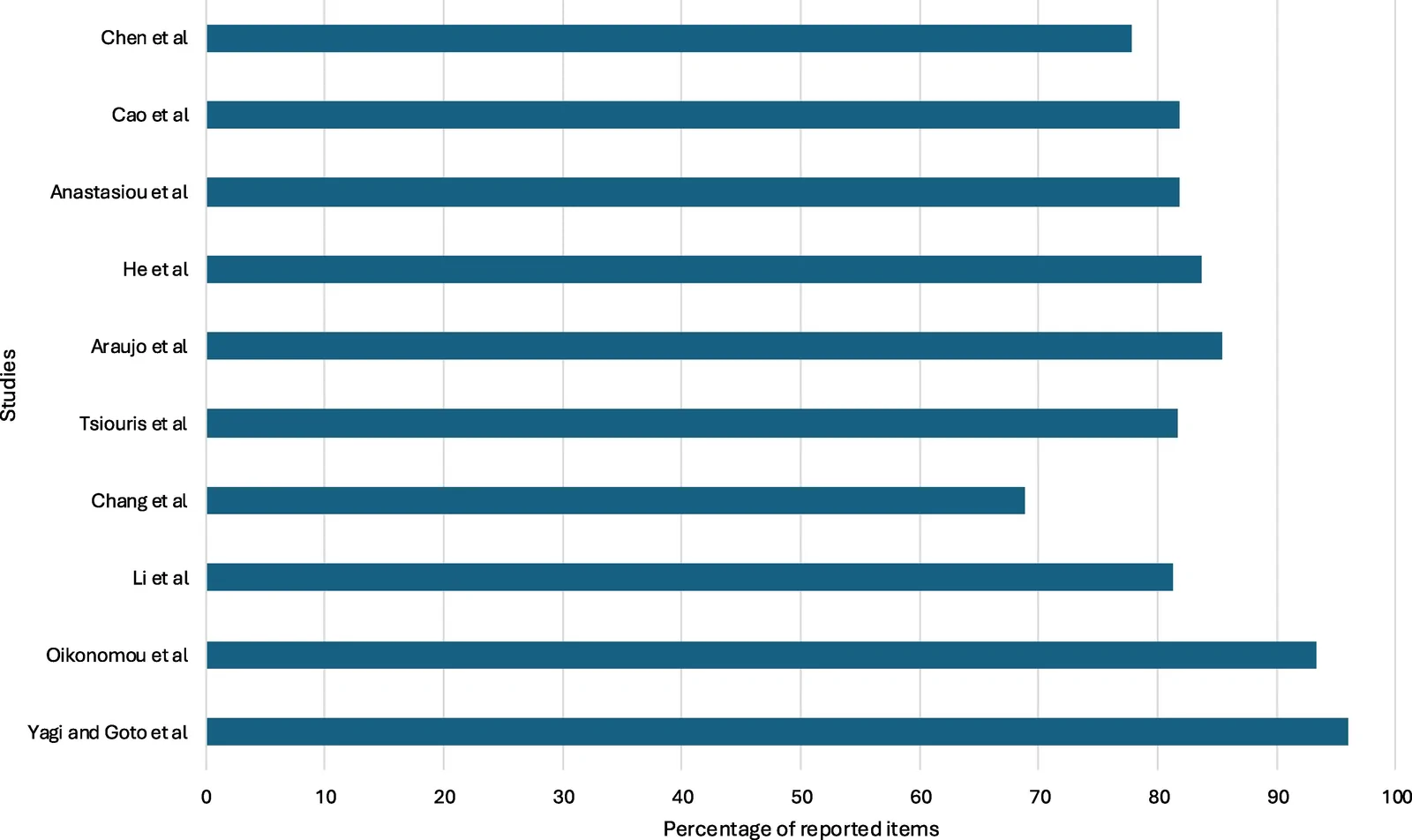
TRIPOD + AI percentage of reported items across studies. The percentages presented in this bar graph are a result of the number of reported items over the number of applicable items from the TRIPO + AI checklist to the particular study.

TRIPOD-AI reporting completeness ranged from 69% to 96% across the nine included studies (*Figure 5*). The most consistently reported items were the study context and rationale, target population and model purpose, study objectives, data sources, and model performance metrics, all of which were addressed in all studies. Similarly, most studies described the selection of initial predictors and the defined model output. The most frequently unreported items were related to sample-size estimation (0% in prospective studies), outcome adjudication procedures and assessor qualifications (reported in only 33% of studies), and calibration metrics (22%); external validation was also absent from most studies (see Supplementary material online, *Table S4*). A formal comparison of the performance of the developed models with the currently used scores was not reported in any of the studies.

## Discussion

This systematic review represents the first comprehensive synthesis of studies that employed diverse ML algorithms to predict CTRCD. By consolidating evidence from nine independent investigations involving 28 ML algorithms, this work provides key insights into the current applicability of these models. Firstly, studies using multiparametric models integrating clinical, biochemical, and imaging data reported superior discriminative performance metrics compared with studies using ECG-only models. The observed improvement in the performance of an ECG-based model after incorporating clinical characteristics further supports this finding. Nevertheless, a superiority of one type of model against another cannot be claimed by this review, since a head-to-head formal comparison has not been performed, and thus should be considered hypothesis-generating. Second, a common challenge across all studies was the relatively low number of events, consistent with the real-world incidence of moderate and severe CTRCD,^3,4,25^ leading to class imbalance that can ultimately compromise the performance of ML-based classification models. Finally, most studies were at high risk of bias, did not report calibration metrics, and lacked external validation, substantially limiting confidence in the real-world applicability and generalizability of these models. Notably, the only two studies that reported calibration metrics were among the most recently published, suggesting a likely shift towards more rigorous methodological and reporting practices in this emerging field.^23,24^ It is important to note that most of the available evidence is derived from patients treated with anthracyclines and anti-HER2 therapies. These agents are widely used in both solid tumours and haematological malignancies, and their association with cardiac dysfunction has long been recognized as a significant limitation to their clinical use.^3,26^ Consequently, the findings of the present review should be interpreted within this specific therapeutic context.

The absence of a reliable tool to identify patients at risk of developing cardiac toxicity represents a significant barrier to the quality of care for cancer patients. A proactive approach that includes a baseline risk assessment provides an opportunity to take preventive action to mitigate CV risk and optimize the use of medical resources. Preventative actions range from CV risk factor optimization and referral to cardio-oncology for investigation and treatment of underlying cardiovascular disease to reconsideration of oncology therapy options when the risks associated with the intended therapy may outweigh the benefits.

Baseline risk assessment is recommended by leading onco-haematology and cardio-oncology medical bodies to guide cardiac monitoring during and after cancer therapy.^7,27^ However, there is a disconnect between these recommendations and real-world practice. One study included in this review reported that approximately 50% of patients at risk had not undergone baseline echocardiography, and 42% lacked follow-up imaging.^17^ Similar findings have been observed in other large cohorts, in which nearly half of patients undergoing systemic therapy did not receive echocardiographic monitoring.^28–30^ This lack of adherence highlights the need for more tailored, facile, and risk-adapted cardiac monitoring strategies that optimize resource utilization, reduce unnecessary imaging, and minimize the likelihood of missed diagnoses.

This review addresses this need by analysing the existing evidence on the performance and applicability of ML-enhanced risk prediction models. The reason for a focused exploration of more novel modalities for risk prediction is that this field was driven by the evidence showing that traditional CV risk scores, which have not been originally designed for this task, tend to systematically underestimate risk in oncology patients,^10^ and existing cancer-specific models based on conventional statistical methods are of questionable quality and prone to bias.^8^ An exception to this would be the HFA-ICOS risk stratification position paper.^6^ The models presented in this document have been validated on different cohorts showing an acceptable level of performance; however, in most cases, the cohorts examined were relatively small and unicentric, thereby limiting the generalizability of their findings.^4,31,32^ A more recent study validated the HFA-ICOS anthracycline risk model in a multicentric cohort of 1066 patients.^25^ The score demonstrated good predictive performance (AUC:0.78, 95% CI 0.70–0.82) for predicting moderate-to-severe CTRCD at 12 months. A major limitation of this validation cohort was the low incidence of patients with the primary outcome (*n* = 68), which may limit the robustness of the findings, according to the TRIPOD statement.^33^ Furthermore, a meta-analysis of studies validating the HFA-ICOS model in patients treated with HER2 targeted therapies (*n* = 5) reported a pooled c-statistic of 0.6 (poor to moderate discrimination) with moderate heterogeneity (*I*^2^: 57.88% (95% CI 0.00–97.40).^9^

In view of the described limitations of existing models, the present review explored the potential use of machine learning for risk prediction in cardio-oncology, highlighting strengths, limitations and potential areas for future refinement in ML-based CTRCD prediction.

Studies based on clinical predictors reported good discrimination metrics; however, they were methodologically constrained. Most models were trained on retrospective data or small, prospective single-centre cohorts with ill-defined inclusion criteria and no formal sample size calculation. We also observed that models were trained on large numbers of predictive variables in populations with low event rates. Three studies applied data augmentation techniques to address this limitation.^21,22,24^ While this is an accepted method to mitigate imbalanced datasets, the high risk of patient-selection bias in some studies and the lack of external validation in almost all studies may have increased the risk of overfitting, thereby limiting generalisability. Nevertheless, an important finding across these investigations was the consistent identification of hypertension and baseline LVEF as key predictors that substantially impact model outputs. Age was only identified among the most influential variables in two studies. Given the well-established association between advancing age and cardiovascular risk, this finding is noteworthy. It is important to note that this variable was surprisingly not included as a predictor in all studies and that one study found that although age showed a significant association with the outcome in univariable analyses, it was not consistently retained as a highly influential predictor when performing the Global Feature Importance and SHAP analysis.^24^ Furthermore, only two studies incorporated previous radiotherapy exposure into its prognostic model. Given that patients with breast cancer and lymphoma frequently receive multimodal treatment—including chemotherapy, radiotherapy, and surgery—and that radiotherapy is associated with potential local cardiotoxic effect,^34,35^ failure to account for this exposure may have limited the predictive performance of these models. In this review, we could identify that predictor selection strategies varied considerably across studies, ranging from data availability, literature-based evidence, and expert opinion to ML-driven feature selection approaches. Chen *et al*.^23^ applied seven different feature selection methods (SR-FS, SR-BS, SR-BE, LASSO, SVM-RFE, RF-RFE, and OSR) and identified five predictors that were consistently selected across methods, including age, baseline LVEF, anthracycline dose, trastuzumab combination therapy, and ECG abnormalities.^23^ Such approaches may offer a more systematic and reproducible framework for predictor selection in future ML-based cardio-oncology studies.

*ECG-based models* demonstrated more robust model design, with one study classified as having low risk of bias and the other as unclear. Both used multicentre registry datasets including more than 1000 patients and algorithms previously developed and validated in different cohorts for related purposes. Importantly, the only required input is a standard 12-lead ECG, which is readily available in most clinical settings, and the risk-assessment tool used in one study is openly accessible to end-users,^16^ unlike other models discussed. This substantially enhances the usability of *ECG-based models*. The main concern in one study was the absence of follow-up echocardiography in 42% of patients, introducing uncertainty regarding outcome adjudication.^17^ Despite the described strengths relative to *clinical predictor-based models*, *ECG-based models* reported lower discrimination metrics. Interestingly, when one of the *ECG-based models* was combined with conventional cardiovascular risk factors, the AUC improved significantly,^17^ underscoring the importance of integrating clinical context rather than relying on isolated signals.

Taken together, these methodological considerations do not diminish the contribution of existing literature but instead emphasize the need for more standardized outcome definitions, larger and more representative cohorts, and routine calibration and external validation to support the development of robust, generalizable ML models for CTRCD baseline risk prediction.

### Future directions

The enthusiasm surrounding ML has generated considerable optimism about its potential applications in cardio-oncology, and this systematic review lays the groundwork for future efforts to develop, validate, and implement robust prediction models in clinical practice. Nonetheless, the current evidence base has limitations in data quality and availability, which significantly affect model performance. Given that machine learning excels in large-scale, high-dimensional datasets, its advantage is diminished in this context.^36^ As such, future research may be better served by focusing on traditional statistical modelling techniques, either by developing new risk scores or recalibrating existing ones, which are often more robust in low-event settings and better suited to small or imbalanced datasets.^36^ An additional priority should be the establishment of large cardio-oncology registries to improve standardized data collection for analysis, thereby supporting the development of more accurate, prospectively validated, and clinically applicable ML-based risk scores. Another important area for improvement in future studies identified by this review is the adoption of standardized outcome definitions, such as the CTRCD definition proposed by ICOS,^2^ and the transparent reporting of outcome adjudication. Furthermore, a systematic comparison of ML-based model performance with currently used risk scores such as the HFA-ICOS risk score is warranted, although this score is primarily based on expert consensus and not derivation-validated. Additionally, there should be a more comprehensive incorporation of treatment-related exposures, including previous radiotherapy, given its recognized potential contribution to cardiovascular toxicity. Finally, the clinical applicability of these models requires further work to assess their utility and acceptability among clinicians. This includes integrating them into accessible platforms for the broader medical community, alongside prospective evaluation of their acceptance, real-world adoption, and opportunities for further refinement.

### Strengths and Limitations

This is the first systematic review to comprehensively examine and delineate machine learning-based risk prediction models for CTRCD, an area which is emerging in the field of cardio-oncology. A significant strength of this review lies in its comprehensive search strategy and strict adherence to PRISMA guidelines, which ensured methodological rigour and transparency throughout. The inclusion criteria were deliberately designed to capture ML-based risk prediction models applied at baseline.

Nonetheless, several limitations must be acknowledged. The number of eligible studies was limited, and substantial heterogeneity in model design, input variables, outcome definitions and adjudication methods, and reported performance metrics, many lacking data on the variance of outcome measures, precluded meta-analysis. However, this reflects the current state of ML-enabled risk prediction in this field and should prompt efforts for further research. Furthermore, the search was restricted to studies published in English, although only two studies were excluded on that basis. Finally, while we did not search machine learning-specific repositories or grey literature, our targeted focus on clinically relevant databases (EMBASE and MEDLINE), supplemented by manual reference screening, makes it unlikely that key eligible studies were missed. Notably, only two additional studies were identified through manual searching, one of which met the inclusion criteria.

## Conclusion

While ML continues to show significant potential in advancing personalized risk prediction, current models for predicting CTRCD remain underdeveloped for clinical use. The present findings emphasize the need for further optimization and validation of current ML models as well as comparative studies of their performance against existing traditional risk scores. Future efforts should also focus on addressing key methodological shortcomings identified in this review, including inadequate calibration assessment, small and unrepresentative datasets, and substantial heterogeneity in outcome definitions and adjudication methods. This will allow the safe and effective translation of ML-based risk prediction models into clinical practice.

## Supplementary Material

ztag127_Supplementary_Data

## Data Availability

No new data were generated or analysed in support of this research
