# Supplementary material for: Machine learning—enhanced baseline risk prediction models for cancer therapy related cardiac dysfunction: a systematic review and critical appraisal of methodological quality
Source: Eur Heart J Digit Health. 2026 Aug 10;7(8):ztag127. doi: 10.1093/ehjdh/ztag127 (PMC13548057; doi:10.1093/ehjdh/ztag127)
Supplement: Supplementary file 1 [file ztag127_supplementary_data.docx]

**Supplementary material**

**Table S1. Search Strategy applied on MEDLINE and EMBASE databases - 21.05.2026**

# **OVID Medline Search:**

| 1) Artificial Intelligence | ("artificial intelligence" or "deep learning" or "exp machine learning" or "neural networks (computer)" or "decision tree" or (artificial adj1 intelligence) or ((deep or machine) adj2 learning) or (vector adj3 machine) or (AI or DL or DLS) or ((deep or convolutional or neural) adj3 network$) or (automat$ adj2 (screen$ or detect$ or diagnos$ or algorithm$ or identif$ or grading or graded or method$)) or (Naive adj1 Bayes) or (Random adj1 Forest) or ((decision or classification or regression or probability or model$) adj3 tree$) or (augment$ adj1 clinical adj1 decision$ adj1 mak$) or (nearest adj1 (neighbor or neighbour))).mp. or kernel.tw. [mp=title, book title, abstract, original title, name of substance word, subject heading word, floating sub-heading word, keyword heading word, organism supplementary concept word, protocol supplementary concept word, rare disease supplementary concept word, unique identifier, synonyms, population supplementary concept word, anatomy supplementary concept word] |
| --- | --- |
| 2) Prediction model | (((predict* or prognos* or (model* or decision* or identif*)) and (Stratification or Discrimination or Discriminate or "c statistic" or "Area under the curve" or AUC or Calibration or Indices or index or Algorithm or Multivariable* or multivariate* or covariate* or valid*)) or (Stratification or Discrimination or Discriminate or "c statistic" or "Area under the curve" or AUC or Calibration or Indices or index or Algorithm or Multivariable* or multivariate* or covariate* or valid*)).mp. [mp=title, book title, abstract, original title, name of substance word, subject heading word, floating sub-heading word, keyword heading word, organism supplementary concept word, protocol supplementary concept word, rare disease supplementary concept word, unique identifier, synonyms, population supplementary concept word, anatomy supplementary concept word] |

| 3) CTRCD | Cardiotoxicity/ or Heart failure/ or (((heart or cardi* or ventricu*) adj1 (disease* or failure or dysfunct* or tox*)) or cardiomyop* or cardiotox* or CTRCD).tw. |
| --- | --- |
| 4) Cancer treatment | ((tumo?r* or neoplas* or cancer* or malignanc* or metasta* or oncolog* or (carcinoma* or lymphoma* or macroglobulinemia or leukemia* or sarcoma* or Hodgkin or glioma* or melanoma* or myeloma* or blastoma*)) and ((h?ematopoietic adj3 "cell transplant*") or ("chemotherapy" or "anthracycline" or "cytotoxic regimens" or "immunotherapy" or "cancer treatment" or "therapy-induced" or "therapy induced" or "targeted therapies" or "targeted adj1 therapies" or "immune checkpoint inhibitor*" or "check point*" or "checkpoint" or "tyrosine kinase inhib*" or TKI or "RET inhib*" or "BRAF inhib*" or (trastuzumab* or deruxtecan or emtansine or hyaluronidase or pertuzumab* or neratinib* or lapatinib* or tucatinib* or dacomitinib* or margetuximab* or enhertu or nerlynx or herceptin or vizimpro or tykerb or tukysa or trazimera or phesgo or perjeta or ontruzant or ogivri or margenza or kanjinti or kadcyla or herzuma)))).mp. [mp=title, book title, abstract, original title, name of substance word, subject heading word, floating sub-heading word, keyword heading word, organism supplementary concept word, protocol supplementary concept word, rare disease supplementary concept word, unique identifier, synonyms, population supplementary concept word, anatomy supplementary concept word] |
| 5) Only humans | exp animals/ not humans.sh. |

Combination: (1 and 2 and 3 and 4) not 5

**OVID Embase Search:**

| 1) Artificial Intelligence | ("artificial intelligence" or "deep learning" or "exp machine learning" or "neural networks (computer)" or "decision tree" or (artificial adj1 intelligence) or ((deep or machine) adj2 learning) or (vector adj3 machine) or (AI or DL or DLS) or ((deep or convolutional or neural) adj3 network$) or (automat$ adj2 (screen$ or detect$ or diagnos$ or algorithm$ or identif$ or grading or graded or method$)) or (Naive adj1 Bayes) or (Random adj1 Forest) or ((decision or classification or regression or probability or model$) adj3 tree$) or (augment$ adj1 clinical adj1 decision$ adj1 mak$) or (nearest adj1 (neighbor or neighbour))).mp. or kernel.tw. [mp=ti, ab, tx, ct, bt, kw, ot, fx, sh, hw, cw, ac, de, md, sd, so, rw, tn, tc, id, tm, mf, pt, an, dm, dv, kf, dq, nm, ox, px, rx, ui, sy, ux, mx] |
| --- | --- |

| 2) Prediction model | (((predict* or prognos* or (model* or decision* or identif*)) and (Stratification or Discrimination or Discriminate or "c statistic" or "Area under the curve" or AUC or Calibration or Indices or index or Algorithm or Multivariable* or multivariate* or covariate* or valid*)) or (Stratification or Discrimination or Discriminate or "c statistic" or "Area under the curve" or AUC or Calibration or Indices or index or Algorithm or Multivariable* or multivariate* or covariate* or valid*)).mp. [mp=ti, ab, tx, ct, bt, kw, ot, fx, sh, hw, cw, ac, de, md, sd, so, rw, tn, tc, id, tm, mf, pt, an, dm, dv, kf, dq, nm, ox, px, rx, ui, sy, ux, mx] |
| --- | --- |
| 3) Cancer treatment | ((tumo?r* or neoplas* or cancer* or malignanc* or metasta* or oncolog* or (carcinoma* or lymphoma* or macroglobulinemia or leukemia* or sarcoma* or Hodgkin or glioma* or melanoma* or myeloma* or blastoma*)) and ((h?ematopoietic adj3 "cell transplant*") or ("chemotherapy" or "anthracycline" or "cytotoxic regimens" or "immunotherapy" or "cancer treatment" or "therapy-induced" or "therapy induced" or "targeted therapies" or "targeted adj1 therapies" or "immune checkpoint inhibitor*" or "check point*" or "checkpoint" or "tyrosine kinase inhib*" or TKI or "RET inhib*" or "BRAF inhib*" or (trastuzumab* or deruxtecan or emtansine or hyaluronidase or pertuzumab* or neratinib* or lapatinib* or tucatinib* or dacomitinib* or margetuximab* or enhertu or nerlynx or herceptin or vizimpro or tykerb or tukysa or trazimera or phesgo or perjeta or ontruzant or ogivri or margenza or kanjinti or kadcyla or herzuma)))).mp. [mp=ti, ab, tx, ct, bt, kw, ot, fx, sh, hw, cw, ac, de, md, sd, so, rw, tn, tc, id, tm, mf, pt, an, dm, dv, kf, dq, nm, ox, px, rx, ui, sy, ux, mx] |
| 4) CTRCD | exp CARDIOTOXICITY/ or exp CARDIOMYOPATHY/ or exp HEART FAILURE/ or exp HEART DISEASE/ or (((heart or cardi* or ventricu*) adj1 (disease* or failure or dysfunct* or tox*)) or cardiomyop* or cardiotox* or CTRCD).tw. |
| 5) Only humans | (animal/ or nonhuman/) not human/ |

Combination: (1 and 2 and 3 and 4) not 5

**Table S2. Adapted data extraction form, based on the CHARMS checklist.**

| **Item** | **Response** |
| --- | --- |
| **Study Characteristics:** |  |
| Author(s): |  |
| Year of publication: |  |
| Journal of publication: |  |
| Study design (e.g., retrospective, prospective, observational, etc.) |  |
| Source of Data: |  |
| **Population Details (Participants):** |  |
| Participant demographics (age, sex) | Age (mean, SD – median, IQR)  Male: |
| Cancer type(s): |  |
| Cancer therapies received (e.g., anthracyclines, HER2 inhibitors, immunotherapy, tyrosine kinase inhibitors): |  |
| Cardiovascular risk factors (hypertension, diabetes, dyslipidaemia, smoker/ex-smoker): | HTN:  DM:  DLP:  SM: |
| Baseline LVEF: |  |
| Exclusion criteria: |  |
| Data collection dates: |  |
| **Outcomes** |  |
| Definition and method for measurement of outcome: |  |
| Type of outcome (‘single’ = CTRCD related alone or ‘combined’ = CVAE in general): |  |
| Time of outcome occurrence or summary of follow-up duration: |  |
| **Predictor variables** |  |
| Predictor variables used in the model (ie: clinical features, echocardiographic features, demographic variables, ECG): |  |
| Number of predictors: |  |
| Timing of predictor measurement (ie: baseline, post treatment, at cancer diagnosis) |  |
| **Sample Size** |  |
| Number of participants |  |
| Number of outcomes/events |  |
| Events per variable (EPV) |  |
| **Data Availability** |  |
| Number of participants with any missing value (include predictors and outcomes) |  |
| Number with missing data for each predictor: |  |
| Handling of missing data (e.g. complete-case analysis, imputation, or other methods) |  |
| **Model development** |  |
| Type of machine learning algorithm (e.g., random forest, support vector machine, neural network): |  |
| Train-Test Split |  |
| Model optimization strategy |  |
| Hyperparameter tuning approach (if applicable) |  |
| **Outcomes: Performance metrics (if reported)** |  |
| - Area under the ROC curve (AUC) |  |
| - Sensitivity |  |
| - Specificity |  |
| - Positive predictive value (PPV) |  |
| - Negative predictive value (NPV) |  |
| - Accuracy |  |
| - Precision |  |
| -F1-Score |  |
| - Calibration (e.g., calibration plots, Brier score) |  |
| Major outcome determinants (if reported) |  |
| **Model Evaluation** |  |
| Method used for performance testing: development dataset only (random split of data, resampling methods e.g. bootstrap or cross-validation, none) or separate external validation (e.g. temporal, geographical, different setting, different investigators) |  |
| If poor validation, was the model updated? □ Yes □ No |  |
| If yes, how was it updated? (e.g., intercept recalibrated, predictor effects adjusted, or new predictors added) |  |
| **Additional Information:** |  |
| Any reported limitations or challenges in the included studies |  |
| Important finding (not reported above) |  |
| Conflicts of interest (if disclosed) |  |
| **Interpretation and Discussion** |  |
| Interpretation of model (confirmatory, i.e., model useful for practice versus exploratory, i.e., more research needed): |  |

**Table S3. Predictors of cardiotoxicity in cancer patients included in prognostic model across studies**

| **Study** | **Clinical predictors** | **Echocardiographic predictors** | **Biochemical data** | **ECG data** |
| --- | --- | --- | --- | --- |
| Chen et al, 2026 | Age  Combined chemotherapy (Anthracyclines + Trastuzumab)  Number of cycles (anthracyclines) | LV ejection fraction |  | Abnormal ECG findings |
| Cao et al, 2026 | Age,  Body mass index,  Hypertension,  Diabetes,  Coronary Artery disease,  TNM stage,  Anthracyclines dose,  Chest radiotherapy,  HER2 targeted therapy  Blood pressure | LV ejection fraction,  LV diastolic diameter  Left atrial diameter  E/A ratio | Liver enzymes,  Renal function tests  Full blood count  Haemoglobin  Haematocrit  D-Dimer  Glucose  Albumin/Glucose ratio | Heart rate  QTc  QRS duration |
| He et al, 2025 | Age,  Body mass index,  Height,  Dyslipidaemia,  Hypertension,  Smoker, Ex-smoker,  Diabetes mellitus,  Time after diagnosis,  Heart rate,  Anthracyclines use,  Previous thorax  Radiotherapy,  Anti-HER2 therapies, | Tissue Doppler Imaging  LV ejection fraction  LV diastolic diameter  Posterior wall thickness  Left atrial diameter  LV fraction shortening |  |  |
| Araujo et al, 2025 | Body mass index,  Weighted smoking status |  | CRP, Total cholesterol, LDLc/HDLc, HDLc, Haemoglobin, Haematocrit |  |
| Tsiouris et al, 2023 | Body Mass Index, Body Surface Area, Gender, Age (at time of therapy),  Medical history of  Hypertension, Diabetes, Hyperlipidaemia,  Smoking, Chronic Kidney Disease,  Cardiomyopathy, Severe Vascular Disease,  Coronary Artery Disease, Congenital Heart  Disease, Arrhythmia, Other Tumour | IVSd, PWd, LVIDd, iLV_Mass, iEDV, LVEF,  GLS, Basal RV, SystRVPress,  TAPSE, LAVI, E, A, E/A Ratio, Deceleration Time,  E' | White Blood Count, Neutrophils,  Haemoglobin, Haematocrit, Platelets, Glucose,  Creatinine, Albumin, liver enzymes Gamma-Glutamyl Transferase,  Alkaline phosphatase, CA, Na, K,  Lactate Dehydrogenase, Total Bilirubin | Heart Rate, LBBB, RBBB,  Abnormal ST segment, Corrected QT interval |
| Chang et al, 2022 | Age, hypertension, Diabetes Mellitus, CAD,  Cancer stage,  Cancer therapies: dose of anthracyclines, use of trastuzumab, taxane, cyclophosphamide,  5-FU, alternative use of epirubicin, operation and radiotherapy | LV ejection fraction at baseline |  |  |
| Li et al, 2022 | Anticancer treatment,  Cancer information,  Demographic variables,  Socioeconomic status,  Prior cardiovascular disease and other comorbidities,  Prior medication |  |  |  |
| Oikonomou et al, 2025 |  |  |  | Baseline ECG (image) |
| Yagi and Goto et al, 2024 |  |  |  | Baseline ECG (image) |

HER2: Human Epidermal Receptor 2, LV: left ventricular, CRP: C-Reactive Protein, LDLc: low-density lipoprotein cholesterol, HDLc: high-density lipoprotein cholesterol, IVSd: Interventricular Septal Thickness in Diastole, PWd: Posterior Wall Thickness in Diastole, LVIDd: Left Ventricular Internal Diameter in Diastole, iLV_Mass: Indexed Left Ventricular Mass, iEDV: Indexed End-Diastolic Volume, LVEF: Left Ventricular Ejection Fraction, GLS: Global Longitudinal Strain, Basal RV: Basal Right Ventricular Diameter, SystRVPress: Systolic Right Ventricular Pressure, TAPSE = Tricuspid Annular Plane Systolic Excursion, LAVI: Left Atrial Volume Index, E: Peak Early Mitral Inflow Velocity (E wave), A: Peak Late Mitral Inflow Velocity (A wave), E/A Ratio: Ratio of Early to Late Mitral Inflow Velocities,
E': Early Diastolic Mitral Annular Tissue Velocity (E prime), LBBB: left bundle branch block, RBBB: right bundle branch block, CAD: coronary artery disease, 5-FU: fluoropyrimidines.

**Table S4. TRIPOD+AI checklist for all studies included**

| Section/Topic | Item | Checklist item | **Chen et al, 2026** | **Cao et al, 2026** | **He et al, 2025** | **Oikonomou et al, 2025** | **Yagi et al, 2024** | **Araujo et al, 2025** | **Tsiouris et al, 2023** | **Chang et al, 2022** | **Li et al, 2022** |
| --- | --- | --- | --- | --- | --- | --- | --- | --- | --- | --- | --- |
| Title | 1 | Identify the study as developing or evaluating the performance of a multivariable prediction model, the target population, and the outcome to be predicted | REPORTED | REPORTED | REPORTED | REPORTED | REPORTED | REPORTED | REPORTED | REPORTED | REPORTED |
| **ABSTRACT** |  |  |  |  |  |  |  |  |  |  |  |
| Abstract | 2 |  | REPORTED | REPORTED | REPORTED | REPORTED | REPORTED | REPORTED | REPORTED | REPORTED | REPORTED |
| **INTRODUCTION** |  |  |  |  |  |  |  |  |  |  |  |
| Background | 3a | Explain the healthcare context (including whether diagnostic or prognostic) and rationale for developing or evaluating the prediction model, including references to existing models | REPORTED | REPORTED | REPORTED | REPORTED | REPORTED | REPORTED | REPORTED | REPORTED | REPORTED |
|  | 3b | Describe the target population and the intended purpose of the prediction model in the context of the care pathway, including its intended users (e.g., healthcare professionals, patients, public) | REPORTED | REPORTED | REPORTED | REPORTED | REPORTED | REPORTED | REPORTED | REPORTED | REPORTED |
|  | 3c | Describe any known health inequalities between sociodemographic groups | NOT REPORTED | NOT REPORTED | NOT REPORTED | REPORTED | REPORTED | REPORTED | REPORTED | REPORTED | REPORTED |
|  | 4 | Specify the study objectives, including whether the study describes the development or validation of a prediction model (or both) | REPORTED | REPORTED | REPORTED | REPORTED | REPORTED | REPORTED | REPORTED | REPORTED | REPORTED |
| METHODS |  |  |  |  |  |  |  |  |  |  |  |
| Data | 5a | Describe the sources of data separately for the development and evaluation datasets (e.g., randomised trial, cohort, routine care or registry data), the rationale for using these data, and representativeness of the data | NOT REPORTED | REPORTED | REPORTED | REPORTED | REPORTED | REPORTED | REPORTED | REPORTED | REPORTED |
|  | 5b | Specify the dates of the collected participant data, including start and end of participant accrual; and, if applicable, end of follow-up | PARTIAL (end of F-U not reported) | PARTIAL (end of F-U not reported) | REPORTED | REPORTED | REPORTED | REPORTED | REPORTED | REPORTED | REPORTED |
| PARTICIPANTS | 6a | Specify key elements of the study setting (e.g., primary care, secondary care, general population) including the number and location of centres | NOT REPORTED | REPORTED | REPORTED | REPORTED | REPORTED | REPORTED | REPORTED | REPORTED | REPORTED |
|  | 6b | Describe the eligibility criteria for study participants | REPORTED | REPORTED | REPORTED | REPORTED | REPORTED | REPORTED | REPORTED | REPORTED | REPORTED |
|  | 6c | Give details of any treatments received, and how they were handled during model development or evaluation, if relevant | REPORTED | REPORTED | REPORTED | REPORTED | REPORTED | REPORTED | REPORTED | REPORTED | REPORTED |
| Data Preparation | 7 | Describe any data pre-processing and quality checking, including whether this was similar across relevant sociodemographic groups | REPORTED | REPORTED | REPORTED | REPORTED | REPORTED | REPORTED | REPORTED | REPORTED | REPORTED |
| Outcome | 8a | Clearly define the outcome that is being predicted and the time horizon, including how and when assessed, the rationale for choosing this outcome, and whether the method of outcome assessment is consistent across sociodemographic groups | NOT REPORTED (time of assessment is not specified) | NOT REPORTED (time of assessment is not specified | REPORTED | REPORTED | REPORTED  (42% of patients had no echocardiogram) | REPORTED | REPORTED | REPORTED | REPORTED |
|  | 8b | If outcome assessment requires subjective interpretation, describe the qualifications and demographic characteristics of the outcome assessors | N/A (used EHR data) | N/A | REPORTED (no information on the level of experience of the sonographers performing the images) | REPORTED | NOT REPORTED (no information on the level of experience of the sonographers performing the images) | NOT REPORTED (no information on the level of experience of the sonographers performing the images) | NOT REPORTED (no information on how the outcome was determined, ie: echo, MUGA, CMR?) | REPORTED | N/A |
|  | 8c | Report any actions to blind assessment of the outcome to be predicted | N/A | N/A | N/A | N/A | N/A | N/A | N/A | N/A | N/A |
| Predictors | 9a | Describe the choice of initial predictors (e.g., literature, previous models, all available predictors) and any pre-selection of predictors before model building | REPORTED | NOT REPORTED | REPORTED | REPORTED | REPORTED | REPORTED | REPORTED | REPORTED | REPORTED |
|  | 9b | Clearly define all predictors, including how and when they were measured (and any actions to blind assessment of predictors for the outcome and other predictors) | REPORTED | REPORTED | REPORTED (all variables were measured prior to treatment – although it does not clarify what time prior to treatment) | REPORTED (Baseline ECG within 12 months before treatment started) | REPORTED (Baseline ECG, within 3 months before treatment) | REPORTED | REPORTED | REPORTED | REPORTED |
|  | 9c | If predictor measurement requires subjective interpretation, describe the qualifications and demographic characteristics of the predictor assessors | N/A | N/A | N/A | N/A | N/A | N/A | N/A | N/A | N/A |
| Sample Size | 10 | Explain how the study size was arrived at (separately for development and evaluation), and justify that the study size was sufficient to answer the research question. Include details of any sample size calculation | REPORTED (the study size was based on data availability, there is no study size calculation) | REPORTED (the study size was based on data availability, there is no study size calculation) | REPORTED (270 P with TDI information, only 27 with event thus employed data augmentation approach to equalize the number of CTTRCD and non-CTRCD to 1:1) | REPORTED (the study size was based on data availability, there is no study size calculation) | REPORTED (the study size was based on data availability, there is no study size calculation) | PARTIALLY/NOT REPORTED (no sample size calculation, no clear explanation of inclusion criteria to arrive to a sample of 78 patients) | REPORTED  (the study size was based on data availability, there is no study size calculation) | NOT REPORTED (no study size calculation) | REPORTED  (the study size was based on data availability, no study size calculation) |
| Missing data | 11 | Describe how missing data were handled. Provide reasons for omitting any data | REPORTED | REPORTED | REPORTED | REPORTED (no missing data) | REPORTED  (no missing data) | REPORTED | REPORTED | REPORTED | REPORTED |
| Analytical methods | 12a | Describe how the data were used (e.g., for development and evaluation of model performance) in the analysis, including whether the data were partitioned, considering any sample size requirements | REPORTED | REPORTED | REPORTED | N/A | REPORTED | REPORTED | REPORTED | REPORTED | REPORTED |
|  | 12b | Depending on the type of model, describe how predictors were handled in the analyses (functional form, rescaling, transformation, or any standardisation). | REPORTED | REPORTED | REPORTED | N/A | REPORTED | REPORTED | REPORTED | NOT REPORTED | REPORTED |
|  | 12c | Specify the type of model, rationale , all model-building steps, including any hyperparameter tuning, and method for internal validation | REPORTED | REPORTED | REPORTED | N/A | REPORTED | REPORTED | REPORTED | REPORTED | REPORTED |
|  | 12d | Describe if and how any heterogeneity in estimates of model parameter values and model performance was handled and quantified across clusters | N/A | N/A | NOT REPORTED | REPORTED | REPORTED | N/A | REPORTED | N/A | NOT REPORTED |
|  | 12e | Specify all measures and plots used (and their rationale) to evaluate model performance (e.g., discrimination, calibration, clinical utility) and, if relevant, to compare multiple models | REPORTED | REPORTED | REPORTED | REPORTED | REPORTED | REPORTED | REPORTED | REPORTED | REPORTED |
|  | 12f | Describe any model updating (e.g., recalibration) arising from the model evaluation, either overall or for particular sociodemographic groups or settings | N/A | N/A | NOT REPORTED | N/A | REPORTED (The study describes model updating through a transfer learning approach) | NOT REPORTED | NOT REPORTED | NOT REPORTED | NOT REPORTED |
|  | 12g | For model evaluation, describe how the model predictions were calculated (e.g., formula, code, object, application programming interface) | REPORTED | REPORTED | REPORTED | REPORTED | REPORTED | REPORTED | REPORTED | REPORTED | REPORTED |
| Class imbalance | 13 | If class imbalance methods were used, state why and how this was done, and any subsequent methods to recalibrate the model or the model predictions | REPORTED | N/A | REPORTED (data augmentation) | REPORTED | REPORTED | REPORTED | REPORTED | NOT REPORTED | REPORTED |
| Fairness | 14 | Describe any approaches that were used to address model fairness and their rationale | REPORTED | REPORTED | REPORTED | REPORTED | REPORTED | REPORTED | REPORTED | NOT REPORTED | NOT REPORTED |
| Model Output | 15 | Specify the output of the prediction model (e.g., probabilities, classification). Provide details and rationale for any classification and how the thresholds were identified | REPORTED | REPORTED | REPORTED | REPORTED | REPORTED | REPORTED | REPORTED | REPORTED | REPORTED |
| Training versus evaluation | 16 | Identify any differences between the development and evaluation data in healthcare setting, eligibility criteria, outcome, and predictors | REPORTED | REPORTED | REPORTED | REPORTED | REPORTED | REPORTED | REPORTED | NOT REPORTED | REPORTED |
| Ethical approval | 17 | Name the institutional research board or ethics committee that approved the study and describe the participant-informed consent or the ethics committee waiver of informed consent | REPORTED | REPORTED | REPORTED | REPORTED | REPORTED | REPORTED | REPORTED | REPORTED | REPORTED |
| OPEN SCIENCE |  |  |  |  |  |  |  |  |  |  |  |
| Funding | 18a | Give the source of funding and the role of the funders for the present study | REPORTED | REPORTED | REPORTED | REPORTED | REPORTED | REPORTED | REPORTED | REPORTED | REPORTED |
| Conflicts of interest | 18b | Declare any conflicts of interest and financial disclosures for all authors | REPORTED | REPORTED | REPORTED | REPORTED | REPORTED | REPORTED | REPORTED | REPORTED | REPORTED |
| Protocol | 18c | Indicate where the study protocol can be accessed or state that a protocol was not prepared | NOT REPORTED | NOT REPORTED | NOT REPORTED | NOT REPORTED | REPORTED | NOT REPORTED | NOT REPORTED | NOT REPORTED | NOT REPORTED |
| Registration | 18d | Provide registration information for the study, including register name and registration number, or state that the study was not registered | NOT REPORTED | NOT REPORTED | NOT REPORTED | NOT REPORTED | REPORTED | REPORTED | REPORTED (Project code: 82863, MIS: 5047133) | NOT REPORTED | NOT REPORTED |
| Data sharing | 18e | Provide details of the availability of the study data | REPORTED | REPORTED | REPORTED | REPORTED | REPORTED | REPORTED | REPORTED | NOT REPORTED | REPORTED |
| Code sharing | 18F | Provide details of the availability of the analytical code | NOT REPORTED | NOT REPORTED | REPORTED | REPORTED | REPORTED | REPORTED | NOT REPORTED | NOT REPORTED | NOT REPORTED |
| PATIENT & PUBLIC INVOLVEMENT |  |  |  |  |  |  |  |  |  |  |  |
| Patient and Public involvement | 19 | Provide details of any patient and public involvement during the design, conduct, reporting, interpretation, or dissemination of the study or state no involvement | NOT REPORTED | NOT REPORTED | NOT REPORTED | NOT REPORTED | NOT REPORTED | NOT REPORTED | NOT REPORTED | NOT REPORTED | NOT REPORTED |
| RESULTS |  |  |  |  |  |  |  |  |  |  |  |
| Participants | 20a | Describe the flow of participants through the study, including the number of participants with and without the outcome and, if applicable, a summary of the follow-up time. A diagram may be helpful. | REPORTED | REPORTED | REPORTED | REPORTED | REPORTED | REPORTED | REPORTED | REPORTED | REPORTED |
|  | 20b | Report the characteristics overall and, where applicable, for each data source or setting, including the key dates, key predictors (including demographics), treatments received, sample size, number of outcome events, follow-up time, and amount of missing data. A table may be helpful. Report any differences across key demographic groups. | PARTIAL (follow-up time not reported) | PARTIAL (follow-up time not reported) | REPORTED | REPORTED | REPORTED | REPORTED | REPORTED | REPORTED | REPORTED |
|  | 20c | For model evaluation, show a comparison with the development data of the distribution of important predictors (demographics, predictors, and outcome) | REPORTED | REPORTED | REPORTED | REPORTED | REPORTED | REPORTED | REPORTED | NOT REPORTED | REPORTED |
| Model development | 21 | Specify the number of participants and outcome events in each analysis (e.g., for model development, hyperparameter tuning, model evaluation) | REPORTED | REPORTED | REPORTED | REPORTED | REPORTED | REPORTED | REPORTED | REPORTED | REPORTED |
| Model specification | 22 | Provide details of the full prediction model (e.g., formula, code, object, application programming interface) to allow predictions in new individuals and to enable third-party evaluation and implementation, including any restrictions to access or re-use | REPORTED | NOT REPORTED | REPORTED | REPORTED | REPORTED | REPORTED | NOT REPORTED | NOT REPORTED | NOT REPORTED |
| Model performance | 23a | Report model performance estimates with confidence intervals, including for any key subgroups | REPORTED | REPORTED | REPORTED | REPORTED | REPORTED | REPORTED | REPORTED | REPORTED | REPORTED |
|  | 23b | If examined, report results of any heterogeneity in model performance across clusters. | N/A | N/A | N/A | REPORTED | REPORTED | NOT REPORTED | NOT REPORTED | NOT REPORTED | REPORTED |
| Model updating | 24 | Report the results from any model updating, including the updated model and subsequent performance | N/A (no model updating performed) | N/A (no model updating performed) | REPORTED (TDI alone, vs TDI w/clinical data and LV function) | N/A (no model updating performed) | REPORTED | N/A (no model updating performed) | N/A (no model updating performed) | N/A (no model updating performed) | N/A (no model updating performed) |
| DISCUSSION |  |  |  |  |  |  |  |  |  |  |  |
| Interpretation | 25 | Give an overall interpretation of the main results, including issues of fairness in the context of the objectives and previous studies | REPORTED | REPORTED | REPORTED | REPORTED | REPORTED | REPORTED | REPORTED | REPORTED | REPORTED |
| Limitations | 26 | Discuss any limitations of the study (such as a non-representative sample, sample size, overfitting, missing data) and their effects on any biases, statistical uncertainty, and generalizability | REPORTED | REPORTED | REPORTED | REPORTED | REPORTED | REPORTED | REPORTED | REPORTED | REPORTED |
| Usability of the model in the context of current care | 27a | Describe how poor quality or unavailable input data | REPORTED | REPORTED | REPORTED | REPORTED | REPORTED | REPORTED | REPORTED | REPORTED | REPORTED |
|  | 27b | Specify whether users will be required to interact in the handling of the input data or use of the model, and what level of expertise is required of users | REPORTED | REPORTED | NOT REPORTED | REPORTED | REPORTED | NOT REPORTED | NOT REPORTED | NOT REPORTED | NOT REPORTED |
|  | 27c | Discuss any next steps for future research, with a specific view to applicability and generalizability of the model | REPORTED | REPORTED | REPORTED | REPORTED | REPORTED | REPORTED | REPORTED | REPORTED | REPORTED |
| NOT REPORTED ITEMS/ APPLICABLE ITEMS |  |  | **10/45** | **8/44** | **8/49** | **3/45** | **2/50** | **7/48** | **9/49** | **15/48** | **9/48** |
| REPORTED/APPLICABLE (N,%) |  |  | **35/45**  **(77.8)** | **36/44**  **(81.8)** | **41/49 (83.6)** | **42/45 (93.3)** | **48/50 (96)** | **41/48 (85.4)** | **40/49 (81.6)** | **33/48 (68.8)** | **39/48**  **(81.3)** |
